# Hemorrhage promotes inflammation and myocardial damage following acute myocardial infarction: insights from a novel preclinical model and cardiovascular magnetic resonance

**DOI:** 10.1186/s12968-017-0361-7

**Published:** 2017-07-04

**Authors:** Nilesh R. Ghugre, Mihaela Pop, Reuben Thomas, Susan Newbigging, Xiuling Qi, Jennifer Barry, Bradley H. Strauss, Graham A. Wright

**Affiliations:** 1 Physical Sciences Platform, Sunnybrook Research Institute, 2075 Bayview Avenue, Room M7-510, Toronto, ON M4N 3M5 Canada; 2 Department of Medical Biophysics, University of Toronto, Toronto, ON Canada; 3 Schulich Heart Research Program, Sunnybrook Health Sciences Centre, Toronto, ON Canada; 4 The Toronto Centre for Phenogenomics, Mount Sinai Hospital, Toronto, ON Canada

**Keywords:** Myocardial infarction, Hemorrhage, Inflammation, Microvascular obstruction, T2, T2*, Ischemia reperfusion injury, cardiovascular magnetic resonance

## Abstract

**Background:**

Myocardial hemorrhage is a frequent complication following reperfusion in acute myocardial infarction and is predictive of adverse outcomes. However, it remains unsettled whether hemorrhage is simply a marker of a severe initial ischemic insult or directly contributes to downstream myocardial damage. Our objective was to evaluate the contribution of hemorrhage towards inflammation, microvascular obstruction and infarct size in a novel porcine model of hemorrhagic myocardial infarction using cardiovascular magnetic resonance (CMR).

**Methods:**

Myocardial hemorrhage was induced via direct intracoronary injection of collagenase in a novel porcine model of ischemic injury. Animals (*N* = 27) were subjected to coronary balloon occlusion followed by reperfusion and divided into three groups (*N* = 9/group): 8 min ischemia with collagenase (+HEM); 45 min infarction with saline (I-HEM); and 45 min infarction with collagenase (I+HEM). Comprehensive CMR was performed on a 3 T scanner at baseline and 24 h post-intervention. Cardiac function was quantified by cine imaging, edema/inflammation by T2 mapping, hemorrhage by T2* mapping and infarct/microvascular obstruction size by gadolinium enhancement. Animals were subsequently sacrificed and explanted hearts underwent histopathological assessment for ischemic damage and inflammation.

**Results:**

At 24 h, the +HEM group induced only hemorrhage, the I-HEM group resulted in a non-hemorrhagic infarction, and the I+HEM group resulted in infarction and hemorrhage. Notably, the I+HEM group demonstrated greater hemorrhage and edema, larger infarct size and higher incidence of microvascular obstruction. Interestingly, hemorrhage alone (+HEM) also resulted in an observable inflammatory response, similar to that arising from a mild ischemic insult (I-HEM). CMR findings were in good agreement with histological staining patterns.

**Conclusions:**

Hemorrhage is not simply a bystander, but an active modulator of tissue response, including inflammation and microvascular and myocardial damage beyond the initial ischemic insult. A mechanistic understanding of the pathophysiology of reperfusion hemorrhage will potentially aid better management of high-risk patients who are prone to adverse long-term outcomes.

## Background

Acute Myocardial Infarction (AMI) occurs due to cessation of coronary blood flow to the heart muscle and the extent of injury follows a wavefront pattern of necrosis in proportion to the duration of occlusion [1]. While prompt reperfusion is favorable for myocardial salvage, ischemia-reperfusion injury (IRI) may often present itself as an adverse consequence. IRI has been associated with expansion of the prior ischemic damage and greater inflammation along with oxidative stress, apoptosis, stunning, free radicals and reperfusion arrhythmias [2, 3]. Lethal IRI may further result in myocardial hemorrhage [4] which, in association with microvascular no-reflow or obstruction (MVO), has been recognized to be a new independent predictor of adverse outcomes [5–8].

The presentation of myocardial hemorrhage as a consequence of IRI has been well documented in both humans [9] and animal models of AMI [10–12]. With recent advances in quantitative Cardiovascular Magnetic Resonance (CMR) techniques, hemorrhage-sensitive relaxation parameters - T2 and T2* have been instrumental in the in-vivo detection of hemorrhagic byproducts [7, 8, 13, 14], and have also made it possible to study the associated long-term consequences. A study by Mather et al. [6] demonstrated that hemorrhage was associated with large infarct size, reduced salvage, greater MVO and lower ejection fraction; it was also the strongest independent predictor of adverse left ventricular (LV) remodeling with an increased risk of arrhythmia and predictive power even greater than MVO. Recently, a much larger study in 286 patients by Carrick et al. [15] has shown that hemorrhage occurred in 40% of ST-segment elevation myocardial infarction (STEMI) patients and that it was independently responsible for adverse remodeling at 6 months. It was further found that hemorrhage was more associated with adverse remodeling and N-terminal pro-brain natriuretic peptide (surrogate outcomes) and cardiovascular death and heart failure following discharge (health outcomes) than MVO.

Although some of these observational clinical studies have reported the prognostic implications of myocardial hemorrhage utilizing the predictive power of CMR, the findings have not been consistent in the literature. Several other investigators have found hemorrhage to only be associated with infarct size and presence of MVO, but not offering prognostic significance beyond these parameters [7, 16]. Given these clinical observations, a systematic and mechanistic understanding of the underlying pathophysiological implications of hemorrhage are not fully understood, particularly as an individual and an additive phenomenon over an ischemic insult.

A critical question is whether hemorrhage is simply a bystander and a marker of severe microvascular injury or does it actively contribute to downstream myocardial and microvascular damage beyond the initial ischemic insult. Furthermore, hemorrhage has always been associated with MVO [6, 17]; however, the interaction and causality between the two is less understood. Our study was designed to probe the pathophysiological consequences of hemorrhage in a large animal model of AMI with CMR as the investigational tool. Our hypothesis was that hemorrhage is a mediator of inflammation, directly contributing to myocardial and microvascular damage in the setting of AMI.

## Methods

### Novel model of hemorrhagic injury

In order to evaluate the contribution of hemorrhage alone in ischemia-reperfusion injury, hemorrhage was artificially induced in porcine hearts by direct intracoronary injection of collagenase. A recent study has demonstrated that intracoronary collagenase is capable of inducing myocardial bleeding in a dose-dependent manner without any mortality attributed to collagenase [18]. This model was modified to study three groups of animals (*N* = 27) subjected to a coronary occlusion: 1) 8 min ischemia with collagenase resulting in only hemorrhage without infarction (+HEM group, *N* = 9) [18]; 2) 45 min ischemia with intracoronary injection of phosphate buffered saline (PBS) resulting in infarction without hemorrhage (I-HEM group, *N* = 9) [19]; and 3) 45 min ischemia with intracoronary injection of collagenase projected to cause infarction with hemorrhage (I+HEM group, *N* = 9).

### Animal protocol

Female Yorkshire pigs (20-25 kg, Caughell Farms, ON) were employed in the study and all procedures were conducted in accordance with protocols approved by the Animal Care Committee of Sunnybrook Research Institute. Before catheterization, animals were sedated with an anesthetic cocktail comprising of atropine (0.05 mg/kg) and ketamine (30 mg/kg). Animals were then intubated, mechanically ventilated and maintained under anesthesia using isoflurane (1-5%). Complete coronary occlusion was achieved distal to the second diagonal branch of the left anterior descending (LAD) artery using an over-the-wire angioplasty balloon dilatation catheter (Sprinter OTW, Medtronic, MN); note that the same level of occlusion was maintained across all animals to ensure consistency. Blood flow was occluded for 8 min (+HEM group) or 45 min (I-HEM and I+HEM groups) and was followed by reperfusion; this was verified by X-ray fluoroscopy (Veradius C Arm, Philips Healthcare). One minute prior to balloon deflation (reperfusion), either 3.2 ml PBS (I-HEM group) or PBS containing collagenase (+HEM and I+HEM groups) (3.2 mg, Clostridium Histolyticum type VII-S, Sigma-Aldrich) was injected through the guide wire port. Cardiac events during ischemia-reperfusion were managed by antiarrhythmic drugs (75 mg over 5 min amiodarone bolus and lidocaine drip as needed) or defibrillation whenever necessary. Animals were then recovered and examined by CMR at 24 h post-intervention and subsequently sacrificed.

### CMR protocol

CMR was performed on a 3 T whole body scanner (MR 750, General Electric Healthcare, Waukesha, Wisconsin, USA) at baseline (healthy state) and at 24 h post-intervention. Cardiac function was evaluated using a steady-state-free-precession (SSFP) sequence in cine mode: 12-15 short-axis slices, TR/TE = 4/1.7 ms, flip angle = 45°, FOV = 24x21.6 cm, matrix = 224x160, slice thickness = 5 mm, 8 views/segment, 20 phases/slice. Edema was quantified using a previously validated T2 mapping sequence [19]; spiral readout with T2 preparation, 10 spirals (12.3 ms, 3072 points), TE = 2.9, 24.3, 45.6, 88.2 and 184.2 ms, 10 short-axis slices, in-plane resolution ~1 mm. Hemorrhage was quantified by T2* mapping using a multi-echo gradient-echo acquisition: 8 echoes (1.4-15.8 ms), TR = 16.8 ms, flip angle = 30°, matrix = 128x128, 10 short-axis slices, in-plane resolution ~1.9 mm. Infarction and MVO were assessed by a T1-weighted inversion recovery gradient echo sequence (IR-GRE) sequence: TR/TE = 4.1/1.9 ms, flip angle = 15°, matrix = 224x192, 2RR intervals, 12-15 short-axis slices, inversion time was adjusted to null the myocardium (250-310 ms). Late gadolinium enhancement (LGE) imaging was initiated at 8-10 min post injection of contrast agent Gadolinium-DTPA (0.2 mmol/kg; Magnevist, Bayer Healthcare).

### CMR data analysis

Cine SSFP images were analyzed using CVI42 software (Circle Cardiovascular Imaging, Calgary, CA) to quantify cardiac function parameters: ejection fraction (EF), end diastolic and end systolic volumes (EDV, ESV) and LV mass. Infarct volume was quantified by segmenting the LGE images using the full-width-half-maximum technique [20] and regions of hypoenhancement within the enhanced infarct were identified with a semi-automatic region growing algorithm using a level set method [21]. Myocardial T2 and T2* maps were generated by pixel-wise fitting with an exponential function using a least squares criterion. Hemorrhage was identified on T2* maps of each short axis slice using the criteria T2* < 20 ms [22] and the corresponding hemorrhage volume was computed. Edema volume was quantified by labeling pixels in the T2 maps whose values were 2 standard deviations (SD) above remote myocardial values [23]; if hemorrhage or MVO were present, then hypointense regions on T2 maps were labeled as edema using the same level set algorithm as mentioned earlier. LGE images and relaxation maps were analyzed using custom-written MATLAB® scripts (The Mathworks, Natick, MA). All volumes were represented as a percentage of the LV volume. Infarct zone T2 and T2* values were also noted in one mid-ventricular slice by copying the segmented infarct regions (from LGE) on to the relaxation maps; if no enhancement was observed on LGE images, then an anteroseptal (LAD) region was chosen. For comparison, baseline and remote myocardial T2 and T2* values were also recorded. Linear regression with goodness of fit analysis was performed between T2 and T2* values to determine relationship between inflammation and hemorrhage.

### Histopathology

Animals were euthanized at 24 h following the CMR examination, hearts were explanted and the LV was sectioned into short-axis slices. Prior to sacrifice, a 4% solution of fluorescent dye thioflavin S (1 ml/kg, Sigma-Aldrich) was intravenously injected as a bolus to examine endothelial cell distribution and arterial patency in the heart [24]. Myocardial slices were immediately placed under an ultraviolet light and photographed to identify regions of no flow represented by Thioflavin S-negative regions. The slices were then immersed into a 1% solution of triphenyltetrazolium chloride (TTC) for 5 min at 37 °C and photographed under room light to identify the infarcted region (TTC-negative). Select slices containing the infarcted tissue were fixed in 10% neutral buffered formalin solution for histological staining. These sections were then dehydrated, embedded in paraffin blocks, sliced into 5 μm thick sections and treated with a) Hematoxylin and eosin (H&E) stain to qualitatively assess gross inflammation, hemorrhage, edema, and necrosis; and b) Immunohistochemistry with macrophage monoclonal antibody MAC387 (Thermo Scientific, Rockford, IL).

### Statistics

All data (cardiac function; infarct, MVO, hemorrhage, and edema volumes; T2 and T2* relaxation parameters) were represented as continuous variables and expressed as mean ± SD. For data involving multiple time points i.e. baseline and 24 h, animal groups were compared using a repeated measures 2-way analysis of variance (ANOVA) statistical analysis with multiple comparisons carried out using the Sidak correction factor (GraphPad Prism®, La Jolla, CA); both within group and across group comparisons were performed. For comparisons across groups only at a single time point (24 h), a one way ANOVA statistical test was performed. Linear regression with goodness of fit analysis was performed between infarct zone T2 and T2* values to determine relationship between inflammation and hemorrhage. A *p* value <0.05 was considered statistically significant.

## Results

### Animal procedures

In the +HEM group, one animal died during the interventional procedure and one animal died post procedure and could not undergo a CMR examination. Two animals died during the infarct procedure in both the I-HEM and I+HEM groups, overall representing a 20% mortality rate associated with the ischemic event. The mortality and exclusions resulted in *N* = 7 animals in each group for further CMR and histological analysis. There were no adverse effects noted or any mortality attributed as a result of the collagenase administration.

### Cardiac function

**Fig. 1:**
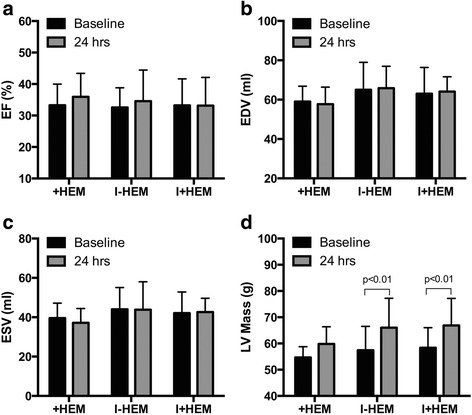
Cardiac Function. *Plots* demonstrate cardiac function parameters - (**a**) ejection fraction (EF), (**b**) end-diastolic volume (EDV), (**c**) end-systolic volume (ESV) and (**d**) left ventricular (LV) mass at *baseline* and 24 h post intervention in the three animal groups. Only LV mass was significantly elevated at 24 h in the 45 min occlusion groups (I-HEM, I+HEM) indicative of inflammatory swelling

Cardiac function parameters by CMR (EF, EDV and ESV) were not significantly different across the three groups at 24 h post intervention (See Fig. 1a-c). However, LV mass was significantly increased at 24 h compared to baseline in the I-HEM (14.9%, *p* < 0.01) and I + HEM (14.5%, *p* < 0.01) groups, indicative of edematous swelling (Fig. 1d). In the + HEM group, mean LV mass was higher at 24 h compared to baseline but did not reach statistical significance (9.5%, *p* = NS).

### Myocardial injury and microvascular obstruction

**Fig. 2:**
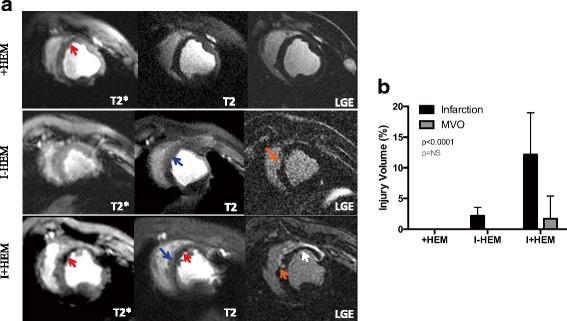
Myocardial Injury and Microvascular Obstruction. **a** Representative T2*-weighted (TE = 16 ms), T2-weighted (TE = 88 ms) and LGE images from a representative animal in each of the three groups, demonstrating the tissue response at 24 h. The *arrows* are indicative of the following pathophysiological features: *Red* – Hemorrhage; *Blue* – Edema; *Orange* – Infarction; and *White* – MVO. Only +HEM and I+HEM groups demonstrated hemorrhage as indicated by low signal intensities on T2* images. The I-HEM group was non-hemorrhagic with a small infarction. High signal intensity on T2 images suggested the presence of edema in the LAD territory in all three groups. No infarction was detected in the hemorrhage only (+HEM) group, but when coupled with an ischemic insult (I+HEM group), it resulted in a large infarction with MVO. **b** *Plot* compares the cumulative infarct and MVO volumes across the three groups

CMR revealed distinct patterns of injury across the three groups of animals at 24 h. Figure 2a shows representative short-axis sections using endogenous T2 and T2* contrast mechanisms beside corresponding LGE images. Hemorrhage was identified by low T2* signal intensity in the anteroseptal region supplied by the LAD coronary artery while edema was described by an elevated T2 signal. The +HEM group demonstrated hemorrhage and some edema but without evidence of infarction or MVO. The I-HEM group was non-hemorrhagic with a small infarction (1.5 ± 1.3 g) and notable edema but no MVO. In contrast, the I+HEM group demonstrated considerable hemorrhage, larger infarction (8.1 ± 4.0 g) and the presence of MVO (1.1 ± 2.5 g; 3 of 7 animals or a 43% occurrence rate). Percent infarct volume was significantly different across the three groups (*p* < 0.0001); see bar plot in Fig. 2b.

### Hemorrhage quantified by T2* mapping

**Fig. 3:**
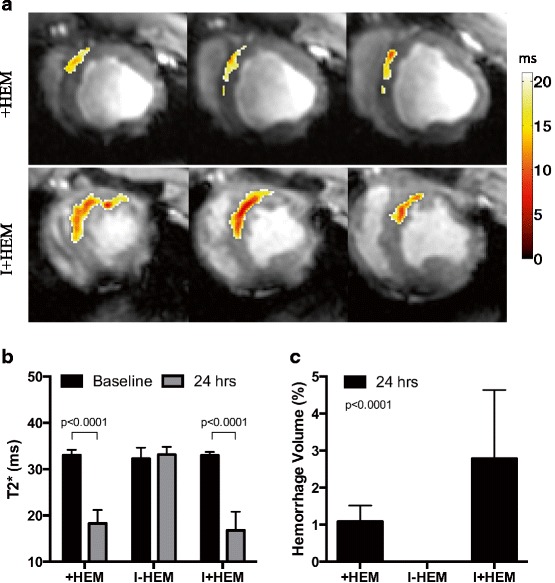
Hemorrhage (T2*). **a** Representative T2*-weighted (TE = 16 ms) images showing a *color overlay* of hemorrhage extent, from animals in the +HEM and I+HEM groups; I-HEM group was non-hemorrhagic. Hemorrhagic sites were segmented using the T2* < 20 ms threshold. Each *row* shows multiple slices from the same animal. **b** Significantly low T2* values in the affected LAD territory in the +HEM and I+HEM groups quantified the occurrence of hemorrhage. **c** Resulting hemorrhage volume was greater when hemorrhage was coupled with an ischemic insult (I+HEM group) as opposed to the hemorrhage only group (+HEM)

Figure 3a shows representative T2* images at 24 h with a color overlay of regions identified as hemorrhage using the T2* < 20 ms criterion. The T2* value in the infarcted/injured territory was significantly depressed in both the +HEM (45%, *p* < 0.0001) and I + HEM (49%, *p* < 0.0001) groups compared to baseline values, indicating the presence of hemorrhage (Fig. 3b). The I-HEM group was non-hemorrhagic with the T2* value statistically unaltered (*p* = NS). Hemorrhage volume based on T2* mapping demonstrated that the extent of hemorrhage was significantly greater in the I+HEM group compared with the +HEM group (2.8 ± 1.8 g vs. 1.1 ± 0.4 g, *p* < 0.0001) (Fig. 3b).

### Edema quantified by T2 mapping

**Fig. 4:**
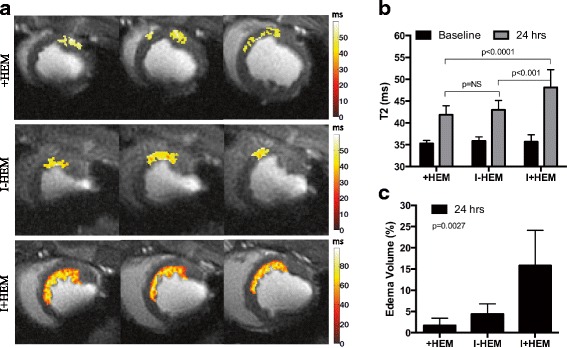
Edema (T2). **a** Representative T2-weighted (TE = 88 ms) images showing a *color overlay* of edema extent, from animals in the three groups. Edematous tissue was segmented using the two standard deviation above remote T2 threshold criteria. Each *row* shows multiple slices from the same animal. Note that edema in the +HEM group was discontiguous (unlike an infarct) potentially due the non-uniform distribution of hemorrhage. **b** *Plot* shows that T2 values were significantly elevated (from baseline) in the affected LAD territory in all three groups, thus quantifying edematous development. **c** *Plot* demonstrates that edema volume was the greatest when hemorrhage and ischemia were coupled together in the I+HEM group

**Fig. 5:**
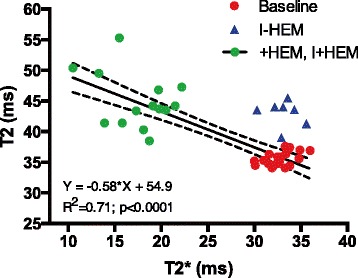
Hemorrhage and Edema. *Plot* demonstrates that the inflammatory response (edema), represented by higher T2 values, was highly correlated with the presence of hemorrhage, represented by lower T2* values. The correlation was tested in animals presenting with myocardial hemorrhage (+HEM, I+HEM groups); non-hemorrhagic animals (I-HEM group) are shown in the *plot* but were excluded in the analysis

Figure 4a shows representative T2 images from the three groups with a color overlay of regions identified as edema (elevated T2) at 24 h. In the +HEM group, intensity of edema in the anteroseptal region, determined by an elevated T2 value, was weak but detectable compared to baseline (19%, *p* < 0.0001). In the I-HEM group, edema in the infarcted tissue was also mild (20% elevation from baseline, *p* < 0.0001) and not significantly different than that in the +HEM group (*p* = NS). The severity of edema in the infarct zone was the greatest in the I+HEM group (35% elevation from baseline, *p* < 0.0001) and was significantly greater than the other two groups (*p* < 0.001) (Fig. 4b). Percent edema volume was also significantly different across the three groups (*p* = 0.0027) and was the greatest in I+HEM (15.8 ± 8.3%) compared to + HEM (1.7 ± 1.7%) and I-HEM (4.4 ± 2.4%) groups (Fig. 4c). In the animals with hemorrhage (+HEM and I+HEM groups), T2 values were highly correlated with T2* values (*Y* = -0.58*X + 54.9; *R* ^*2*^ = 0.71; *p* < 0.0001); see plot in Fig. 5.

### Histopathology

**Fig. 6:**
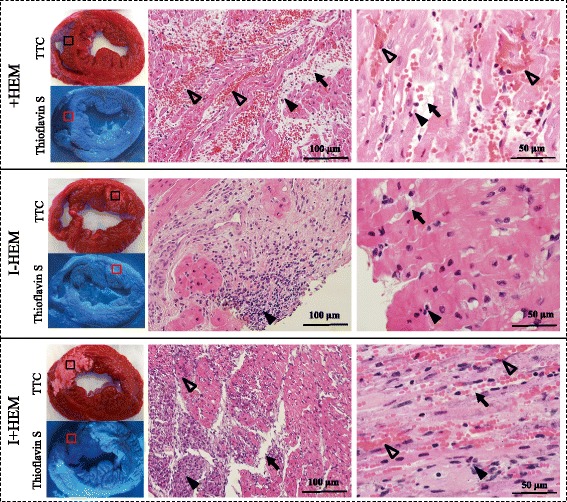
*Injury Patterns* on Histology. Images show TTC, Thioflavin S and H&E stained *short axis* sections obtained from explanted hearts at 24 h post intervention. TTC sections indicate region on necrosis appearing white in the I-HEM group (no hemorrhage) and *reddish white* in I+HEM group (hemorrhage); the +HEM group did no show infarction. Under ultra-violet light, non-fluorescent regions on the Thioflavin S stain highlight areas of compromised endothelium as seen in the +HEM and I+HEM groups; this confirms presence of microvascular damage in the hemorrhage groups. The H&E images were obtained from the region of interest shown on the TTC stained sections. Hemorrhage was apparent in the +HEM and I+HEM groups as evidenced from the interstitial distribution of red blood cells (*open arrow heads*) in the affected LAD territory; red blood cells were absent in the I-HEM group. Edematous development was observed in all three groups (*arrows*) along with the presence of inflammatory cells (*closed arrow heads*)

**Fig. 7:**
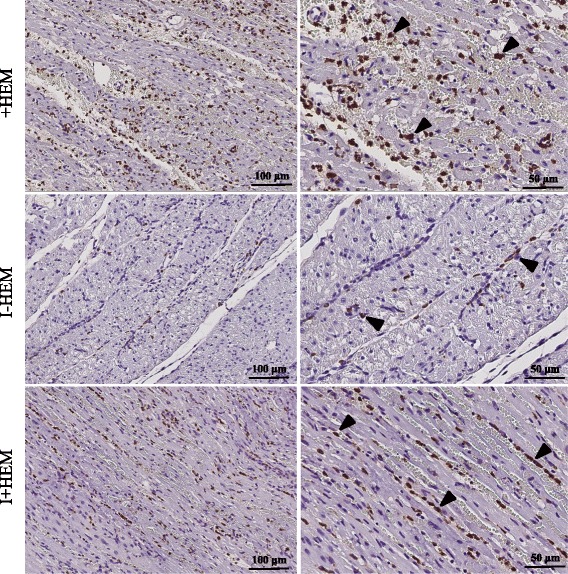
Macrophage Activity. Images show MAC387 macrophage stained sections from the LAD territory. Macrophage infiltration was extensive in both the hemorrhagic groups (+HEM and I+HEM) while it was scarce in the non-hemorrhagic infarct (I-HEM). *Arrowheads* indicate sites with macrophage activity. Notably, macrophages were predominantly concentrated beside the spilled red blood cells in the interstitial spaces. (*Left* column: 20x; *Right* column: 40x)

Figure 6 shows the gross pathology of the heart sections stained with TTC and Thioflavin-S along with a corresponding panel of H&E stained sections from representative animals. In the infarct zone of the +HEM group, myocardium was distended with pools of red blood cells within wide interstitial spaces (edema), and a moderate number of inflammatory cells, particularly lymphocytes and neutrophils. No red blood cells were observed in the infarcted territory of the I-HEM group; however, this region was characterized by necrosis, degenerate myocytes, small interstitial spaces (less edema) and small patchy areas of inflammatory cells. In contrast, the infarcted myocardium of the I+HEM group contained extensive red blood cells, necrosis, wide interstitial spaces (edema), basophilic granular material (calcium) and widespread presence of lymphocytes and macrophages and occasional fibroblasts. In both groups +HEM and I+HEM, sites of hemorrhage were spatially correlated with Thioflavin S-negative regions indicative of endothelial disruption whereas the I-HEM group demonstrated vessel patency in the infarct zone via Thioflavin S-positive signal. Qualitative observations indicated that macrophage staining was scarce and patchy in the non-hemorrhagic I-HEM group (Fig. 7). However, it was moderate to extensive in the +HEM and I+HEM groups. In these two hemorrhagic groups, it was noteworthy that macrophage activity was predominantly concentrated around the dispersed red blood cells.

## Discussion

This is the first study to systematically and mechanistically demonstrate that hemorrhage is a critical component of ischemia-reperfusion injury. The novel preclinical model developed in this study was able to create three distinct patterns of ischemic injury – hemorrhage only, infarction without hemorrhage and infarction with hemorrhage. This offered a means of probing the contribution of myocardial hemorrhage in AMI in a progressive manner. Key findings of our study were as follows: 1) hemorrhage alone can induce a mild inflammatory response in the heart; 2) hemorrhage when combined with a mild ischemic insult, can further aggravate inflammation and result in greater myocardial injury beyond the initial ischemic insult; 3) hemorrhage can contribute towards the occurrence of an MVO; and 4) edema is correlated with the degree of hemorrhage and the hemorrhagic sites are targeted by infiltrating macrophages. Overall, these observations suggest that a significant interaction occurs between hemorrhage, the inflammatory response and ischemic injury. Thus, hemorrhage is not simply a bystander, but an active modulator of cardiac injury and the reparative processes that follow mechanical reperfusion in AMI.

The pathophysiological mechanisms leading to reperfusion hemorrhage in humans are currently unclear. A comprehensive review on intramyocardial hemorrhage in AMI has been recently published by Betgem et al. [25]. In as early as 1960, Jennings et al. [26] identified hemorrhage as one of the key histological features of IRI in a canine model of myocardial infarction; hemorrhage always presented itself within the infarct core [24]. The primary mechanism for the development of hemorrhage appears to be via hypoxia-induced endothelial disruption. The absence of coronary blood flow might lead to increased endothelial stress, swelling and blebbing, further causing loss of interendothelial junction integrity. Beyond ischemia, reperfusion is thought to aggravate the endothelial junction leakiness and damage resulting in extravasation of red blood cells into the tissue interspaces. It has been also been demonstrated that permanent occlusions have resulted only in cellular swelling without hemorrhage and that reperfusion is a necessary condition for the occurrence of hemorrhage [4, 27–29]. Experimental studies have indicated that hemorrhage progressively increases with the duration of coronary occlusion; hemorrhage appears after 50-60 min occlusion in canines [26] and after 45 min occlusion in pigs [10, 19]. Hemorrhage is thus considered a hallmark of severe microvascular damage.

Clinical studies have shown that patients with durations of ischemia >4 h are at significantly high risk of experiencing major adverse cardiovascular events, such as re-infarction, repeat revascularization, heart failure and death [30]. These adverse outcomes appear to be highest in hemorrhagic infarcts [5, 6], although, it is still not clear whether hemorrhage has prognostic significance beyond infarct or MVO size. In a study involving STEMI patients, Beek et al. [16] found hemorrhage to be closely associated with infarct size, presence of MVO and cardiac function, however with no prognostic significance beyond MVO. In a study with reperfused AMI patients, Bekkers et al. [7] further concluded that MVO and hemorrhage together only represent a consequence of a severely damaged microvasculature in large infarction and that only infarct size is an independent predictor of adverse remodeling. Thus, the true role of hemorrhage remains less understood.

To understand the consequences of hemorrhage, animal models have been utilized to show differences between hemorrhagic and non-hemorrhagic infarcts using either fixed or variable occlusion times [14, 19]. For example, in the porcine model, it has been shown that 45 min coronary occlusions result in a non-hemorrhagic infarct [19] and that 45 min appears to be a threshold for the presentation of intramyocardial hemorrhage [10]; hence this model was our choice to serve as the control group. However the presence or absence of hemorrhage in these models is dependent on the physiological response of the animal to ischemia coupled with coronary architecture. Moreover, routine occlusion models cannot assess the effect of hemorrhage alone as they are always associated with ischemic injury. This is the case with clinical studies as well – clinical studies are able to determine outcomes of hemorrhage in association with infarction and MVO but cannot isolate the contribution of hemorrhage post-AMI or assess its active interaction with the healing process. In contrast, our proposed collagenase model offers a more controlled and systematic framework to interrogate the effect of hemorrhage in a progressive manner.

Collagenases are a group of metalloproteinases that can disrupt the interstitial and basal membrane collagen matrix [31]; when injected in a blood vessel this results in an increase in wall permeability and thus spillage of blood into the extravascular space. Collagenase-induced bleeding has been widely used in small animal models of intracerebral hemorrhage and the method has been shown to be reproducible without causing tissue necrosis [32]. Osherov et al. [18] were the first to show the effect of collagenase in the heart. They demonstrated that a single bolus injection of bacterial collagenase into a normal coronary artery was able to induce localized myocardial hemorrhage in a dose-dependent manner. For the +HEM group, we utilized an 8 min occlusion model, similar to that used by Osherov et al., to avoid any backflow of the infusate and to ensure the collagenase effects were confined to the territory of occlusion. At high dose levels, moderate sized hemorrhages were present in both epicardial and intramyocardial tissue. In our study we chose a high dose regimen of 3.2 mg bolus to ensure the consistent occurrence of intramyocadial hemorrhage in all animals. Utilizing this model, the pattern of distribution of myocardial hemorrhage observed in our study closely approximated previously observed changes following a 90 min LAD occlusion model [33].

Although the occurrence of myocardial hemorrhage following AMI has been known from human autopsy studies from the 1980s and preclinical tissue specimens [9, 10, 34], this feature was neglected due to lack of sensitive imaging techniques for in vivo detection. The in vivo identification of hemorrhage with CMR is a relatively new finding, especially in humans. Shortening of T2 and T2* relaxation times have been exploited to detect and quantify hemorrhage degradation products post-AMI [5, 6, 8, 13, 14, 17, 35, 36]; these parameters have further been validated against histopathological ground truth as well as tissue iron stores [14, 17, 35]. Visualization of hemorrhage with CMR has established the link between hemorrhage and compromised function, major cardiac events, re-hospitalization and death [37, 38]. Thus, there has been renewed interest in reperfusion hemorrhage due to its adverse consequences presented in the clinic.

The sole effect of hemorrhage in tissue has been extensively studied in the brain for understanding the implication of intracerebral hemorrhage [39–41]. CMR relaxation parameters (T1, T2, T2*) have also been well characterized in the case of brain hemorrhage along with the alterations during hemorrhagic transformation [42]. Animal models of brain hemorrhage have been instrumental in correlating the effects of hemorrhage alone on inflammation and adverse neurological effects [32]. Our study is the first to utilize this collagenase model to demonstrate that hemorrhage itself may have similar detrimental effects in the heart as well. Our findings indicate that the presence of hemorrhage attracts abundant macrophages (MAC387 positive cells) to the site of injury and interestingly, the recruitment is qualitatively many fold greater than that seen with a mild ischemic insult where macrophage infiltration is scarce, even in the acute setting. This indicates that hemorrhagic byproducts themselves are inherently pro-inflammatory in the heart and that greater hemorrhage (lower T2*) is associated with a larger inflammatory response (higher T2). Note that the low inflammatory status offered by the 45 min occlusion model (I-HEM), as demonstrated earlier [19], provided a reference for the other two groups, with which we could observe that if a small ischemic insult were coupled with hemorrhage, then the inflammatory response is further amplified. This overdriven inflammatory response may possibly be the first-stage mechanism for downstream adverse consequences in hemorrhagic infarcts.

Following successful reperfusion, the ‘no-reflow’ phenomenon is often encountered, characterized by ischemia-induced microvascular obstruction (MVO) and has been correlated with adverse left ventricular (LV) remodeling and poor patient outcome [43]. Recent studies have indicated that the clinical presentation of MVO and myocardial hemorrhage can be as high as 25%-63% in patients with ST-segment elevation myocardial infarction (STEMI) [5–8], and represents a high-risk group. It is well known now that hemorrhage and MVO are related; however, the causal relationship between the two has been less explored. It is uncertain whether MVO causes endothelial damage resulting in blood leakage, making hemorrhage simply a marker of severity, or whether pools of hemorrhagic sites compress the vessels leading to or worsening an MVO. Kumar et al. have demonstrated in a dog model that degree of hemorrhage increases with the size of infarction and also with extent of MVO [44]. Recent findings in STEMI patients by Carrick et al. [15, 45] demonstrate that MVO almost always precedes hemorrhage in the evolution of reperfused ischemic injury. It was shown that the temporal evolution of hemorrhage was different than that of MVO, where MVO volume peaked maximally between 4-12 h post-AMI and decreased thereafter while hemorrhage volume gradually increased from 4-12 h post-AMI, peaked at 72 h and then decreased thereafter. This study concluded that hemorrhage is a downstream consequence of MVO. This study also indicated that it is possible to observe an MVO without hemorrhage in some patients. However, it is important to note that the degree of hemorrhage is highly variable spatially within the MVO region as well as across patients i.e. it is possible to have a very small hemorrhagic region with in a very large MVO. In this regard, Carrick et al. utilized only three T2* slices in their study to detect hemorrhage, which has been acknowledged as a limitation, as a result of which regions of hemorrhage could have been missed in patients identified as having MVO but no hemorrhage.

Hence, mechanistic studies are still lacking, particularly related to complex interaction of hemorrhage with the ischemic tissue bed and vasculature. Specifically, once hemorrhage is present, does it contribute to worsening the ischemic injury and aggravating the MVO (causality)? Our study was designed to address this aspect and we speculate that vascular compression by hemorrhage and the associated inflammatory response (edema) could potentially worsen an MVO. In the + HEM group, we did not observe the classic MVO, however histology confirmed the presence of a perfusion deficit with the Thioflavin S stain. A possible explanation for this could that the regions of perfusion abnormality were too small and subtle to be detected on CMR given that this group did not present with a classical infarct. However, our findings indicate that ischemia in association with hemorrhage (I+HEM group) leads to a greater incidence of MVO suggesting a potential causal relationship between the two. Furthermore, a secondary inflammatory response arising from hemorrhage could also contribute to the no-reflow phenomenon as suggested in the literature [46]. We would also like to note that in the clinical situation, hemorrhage is not seen in the absence of an MVO post-AMI, as observed in animals in the +HEM group and some in the I+HEM group. Our animal model was not aimed to directly replicate the clinical scenario but rather identify the pathophysiological implications of hemorrhage itself, given that it is now confirmed to be a better predictor of adverse outcome than MVO.

We did not note any significant difference in cardiac function parameters, particularly EF at 24 h post-intervention across the three groups. This could potentially be attributed to the relatively small infarct sizes observed in the I-HEM and I+HEM groups with mean infarct size of 2% and 12% (of LV mass) respectively; +HEM group did not show an infarction on CMR. Based on our previous study utilizing a 90 min occlusion model [19], an infarct size greater than 20% might be necessary to see a significant alteration in cardiac function parameters in the early sub-acute phase. Nevertheless, further chronic studies are necessary to evaluate cardiac remodeling as a consequence of the hemorrhage induced by the collagenase model.

### Limitations

We acknowledge several limitations. Firstly, the collagenase-based model for artificially induced hemorrhage is not a true representation of reperfusion hemorrhage resulting from injured/leaky microvasculature. Nevertheless, the model was able to achieve a similar microvascular response. Histological findings clearly identified the extravasation of blood in the interstitial spaces, creating a blood distribution pattern identical to that seen in a 90 min LAD occlusion hemorrhagic model of porcine AMI [17, 19]. Thus the goal of the collagenase model was not to mimic all aspects of reperfusion hemorrhage, but rather to introduce hemorrhage in a manner similar to that seen in IRI, i.e. through injured microvasculature.

Our study also cannot address or distinguish the effects (eg. inflammatory response) of collagenase itself from that arising from hemorrhage, which occurs as a result of its administration; this is an inherent limitation of the model. Nevertheless, the collagenase model does offer an elegant methodology for creating hemorrhagic tissue, similar to that utilized in brain hemorrhage studies [47, 48]. Our findings are in good agreement with the extensive studies investigating collagenase-induced bleeding in the brain; these studies have demonstrated that the features and consequence (edema, inflammation) of this model closely represent that seen in the case of human intracerebral hemorrhage. The collagenase model was able to achieve the intended result of blood spilling into the interstitial spaces of the myocardium. Furthermore, we would like to highlight that irrespective of the other possible effects of collagenase (if any), our study does demonstrate that hemorrhage itself induces an inflammatory response as evidenced by the presence of macrophages near red blood cells under histology, identical to that observed in the collagenase-based studies in the brain [49].

Despite a fixed dose of intracoronary collagenase, the amount of myocardial hemorrhage induced was variable across animals; in some animals hemorrhage was predominantly epicardial with some right ventricular involvement. This would be expected in a cohort of animals since the degree of microvascular response to enzymatic action and ischemia is not predictable. Nevertheless, the selection of a high dose collagenase regimen ensured a consistent induction of myocardial hemorrhage within a given group.

Lastly, T2 measurements can be influenced by both edema and hemorrhage; T2 is elevated in edematous conditions whereas it is lowered by hemorrhage and its by products [17, 33, 42]. Hence our study may have underestimated the intensity and extent of edema, as measured by the T2 parameter, in the hemorrhagic cases. In addition, given the absence of an infarct and uneven distribution of hemorrhage in the +HEM group, the T2-based regions of edema appeared to be non-contiguous given the nature of the injury. Yet, histology patterns agreed well with T2 in representing the inflammatory response with areas of wide interstitial spaces and inflammatory cell aggregation correlating well with regions of elevated T2 values; this has also been verified in our prior porcine study [17].

## Conclusions

Hemorrhage is not simply a bystander but an active contributor to cellular and microvascular damage and inflammation beyond the initial ischemic insult. CMR is a powerful investigational tool that was able to reveal the underlying interactions of hemorrhage and thus demonstrate its critical role in ischemia-reperfusion injury. Future studies can be designed to address the chronic effects of hemorrhage using this novel animal model. A mechanistic understanding of the pathophysiology of reperfusion hemorrhage in the setting of AMI will potentially aid better management of the high-risk patients who are prone to adverse long-term outcomes.

## Abbreviations

+HEM: Hemorrhage without Infarction
AMI: Acute Myocardial Infarction
ANOVA: Analysis of Variance
CMR: Cardiovascular Magnetic Resonance
EDV: End Diastolic Volume
EF: Ejection Fraction
ESV: End Systolic Volume
H&E: Hematoxylin and Eosin
I+HEM: Infarction with Hemorrhage
I-HEM: Infarction without Hemorrhage
IR-GRE: Inversion Recovery Gradient Echo Sequence
IRI: Ischemia-Reperfusion Injury
LAD: Left Anterior Descending coronary artery
LGE: Late Gadolinium Enhancement
LV: Left Ventricle
MVO: Microvascular Obstruction
PBS: Phosphate Buffered Saline
SD: Standard Deviation
SSFP: Steady State Free Precession
STEMI: ST-Segment Elevation Myocardial Infarction
TTC: Triphenyltetrazolium Chloride
